# Further evidence for an HLA-related recessive mutation in nasopharyngeal carcinoma among the Chinese

**DOI:** 10.1038/sj.bjc.6602347

**Published:** 2005-02-22

**Authors:** S P Hu, N E Day, D R Li, R N Luben, K L Cai, T Ou-Yang, B Li, X Z Lu, B A J Ponder

**Affiliations:** 1 Center for Molecular Biology, Shantou University Medical College, Xin Ling Road, Shantou 515031, People's Republic of China; 2 Departments of Public Health and Oncology, Strangeways Research Laboratory, University of Cambridge, Wort's Causeway, Cambridge CB1 8RN, UK; 3 Tumor Hospital, Shantou University Medical College, Rao Ping Road, Shantou 515031, People's Republic of China; 4 Department of Health Care, First Affiliated Hospital, Shantou University Medical College, Chang Ping Road, Shantou 515041, People's Republic of China

**Keywords:** nasopharyngeal cancer, genetic susceptibility, HLA-related genes

## Abstract

We typed 247 cases of nasopharyngeal carcinoma (NPC), a disease predominantly of the southern Chinese, and 274 controls from the Chao Shan region of China's Guangdong province for HLA A and B. Besides confirming the established associations with A2, A33, B46 and B58 (positive associations) and A11 (negative association), the results demonstrated a number of rarer alleles with strong negative association with NPC. Our data, combined with those from the previous studies in Southern Chinese, displayed the protective effects for A31 (odds ratio (OR)=0.0; 95% confidence interval (CI)=0–0.11), B13 (OR=0.50; 95% CI=0.35–0.69), B27 (OR=0.49; 95% CI=0.25–0.92), B39 (OR=0.18; 95% CI=0.06–0.48) and B55 (OR=0.32; 95% CI=0.14–0.68), the ORs comparing individuals with or without each allele. Other ethnic groups do not display such large HLA-associated variation in NPC risk. We show that a linked NPC gene with dominant mode of action could not generate such large protective effects. The results provide strong supporting evidence for the existence of a southern Chinese specific, recessive NPC gene closely linked to the HLA region as a major determinant of the Chinese risk for the disease.

Nasopharyngeal carcinoma (NPC) is largely more common among people originating from southern China than among most other populations in the world. Populations related to the southern Chinese, including the Malays, Thais, Vietnamese and Philippinos, are at intermediate risk (Parkin *et al*, 2002). The hypothesis that there could be a genetic basis for much of this excess risk was strengthened by the identification of a HLA B allele, B46, and associated haplotype A2-B46, which confers increased risk for NPC and is at particularly high frequency among the southern Chinese (Simons *et al*, 1975). The risk attributable to A2-B46, however, accounts for only approximately 20% of the excess NPC risk (Chan *et al*, 1983). A later sib-pair study (Lu *et al*, 1990) demonstrated linkage to the HLA region, suggesting the existence of a recessive NPC susceptibility gene, conferring high risk for NPC and with a gene frequency of 30% or more among the southern Chinese, which could account for much of the excess Chinese risk, although a later linkage study (Feng *et al*, 2002) based on 20 high-risk families (average number of cases per family 3.4) did not confirm this linkage. Subsequent association studies among the Chinese have elaborated further HLA association with NPC, notably, an increased risk associated with B58, and a decreased risk for A11 (Chan *et al*, 1983; Hildesheim *et al*, 2002; Lu *et al*, 2003). Among other ethnic groups, however, the pattern of association with HLA alleles is rather different, with A2 appearing protective in Caucasians (Burt *et al*, 1994), and A11 not conferring protection among north Africans (Betuel *et al*, 1975; Herait *et al*, 1983; Dardari *et al*, 2001). The present study was undertaken to examine further the HLA A and B associations among southern Chinese.

## MATERIALS AND METHODS

### Study population

The study subjects for both cases and controls were restricted to those who were born and whose families had resided in Chao Shan region of Guangdong Province, China, an area of high NPC prevalency (Parkin *et al*, 2002), for more than two generations. Nasopharyngeal carcinoma patients were identified at their initial visit between January 2001 and April 2004 for a nasopharyngeal examination in the Tumor Hospital, Shantou University Medical College, China. Peripheral blood was taken from 247 patients in whom the diagnosis of NPC was pathologically confirmed. Controls were randomly selected from those who had their physical check-up in the first affiliated hospital of the same university, and blood drawn from 274 unrelated healthy individuals with no family and personal history of cancer. Besides, familial members of 25 NPC patients, including spouse, at least one child, and/or one of the patient's parents, were recruited to confirm the validity of the typing results. The average ages of cases and controls were 47.6 years (range 14–76 years) and 38.0 years (range 10–77 years), respectively, with 79% (196 out of 247) of patients and 43.1% (118 out of 274) of controls being ⩾40 years old. The male : female ratio was approximately 3 : 1 (186 : 61) in the case group and 1.3 : 1 (158 : 118) in the control group. Informed consent was obtained from the study participants. The study was approved by the ethical review committees of the appropriate institutions.

### HLA genotyping

DNA was extracted from tri-sodium citrate anticoagulant blood using DNA Blood Mini Kit (Watson, Shanghai, China) following the manufacturer's instructions. HLA class I genes (HLA A, B) were typed following the PCR-SSP method as described (Bunce *et al*, 1995; Bunce, 2003) with some modifications. Briefly, all of the primers for HLA A and B typing as listed in Table 3a and 3b (Bunce *et al*, 1995) were used, except those in lanes 24, 29, 30, 35, 44, 48, 52, 72, and 74. The control primers giving rise to a 796-bp fragment from the third intron of HLA-DRB1 were included in all the PCR reactions. PCR reactions were set in 0.2-ml 96-well PCR plates (Abgene, Epsom, UK) in 10 *μ*l of 1 × PCR reaction buffer, overlaid with 10 *μ*l of mineral oil, containing 45 ng DNA, 200 *μ*M of each dNTP, 0.5 and 0.1 *μ*M of each allele-specific and control primers, respectively, 0.25 U *Taq* DNA polymerase (Promega Shanghai, China), 0.1% Ficoll-400, and 0.1 *μ*g cresol red. The amplifications were carried out in a PTC-225 Tetrad machine (MJ Research, USA) with the cycling parameters of 2 min at 96°C followed by five cycles of 25 s at 96°C, 1 min at 70°C, 1 min at 72°C, then 21 cycles of 25 s at 96°C, 50 s at 65°C, 50 s at 72°C, and final four cycles of 25 s at 96°C, 1 min at 55°C, 2 min at 72°C. Fragment sizes were analysed by 2% agarose gel electrophoresis and visualised using the Gel Doc 2000 system (Bio-Rad, USA).

### Statistical analysis

The proportion of individuals carrying each HLA A and B allele was computed for cases and controls separately. Differences in proportions between cases and controls were expressed as odds ratios (ORs). Confidence intervals (CIs) and the statistical significance of departures from unity of the ORs were computed using standard techniques for 2 × 2 tables, or combinations of 2 × 2 tables when data from several studies were combined, including exact methods where appropriate (Breslow and Day, 1980), by means of STATA 8. Age and sex were available, but their inclusion in the analysis made negligible difference. The results are presented without age and sex adjustment, as neither is related to HLA frequencies; and their inclusion would be formally inappropriate (Breslow and Day, 1980).

## RESULTS

**Figure 1:**
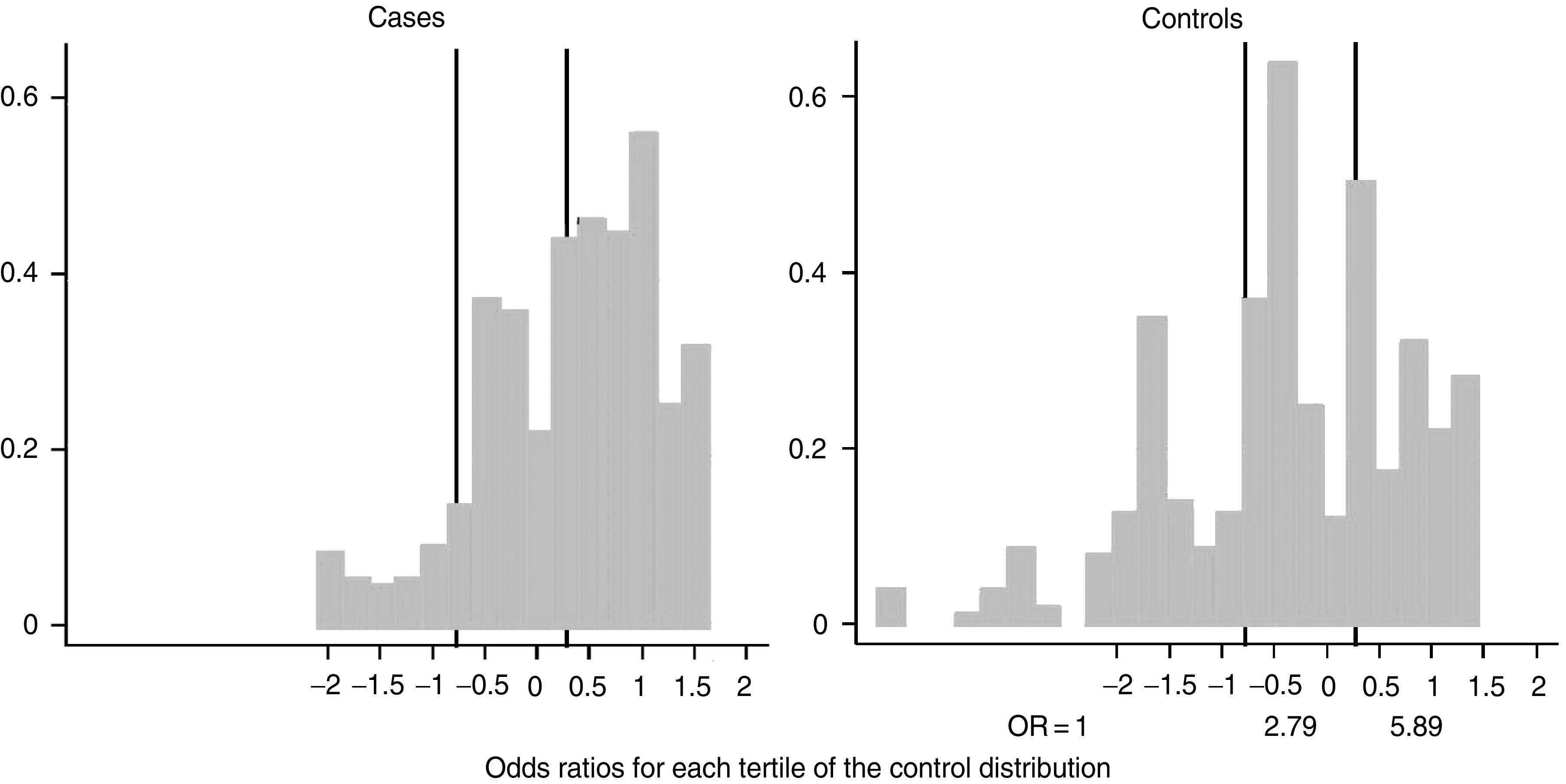
Distributon among cases and controls of a score calculated for each individual as the sum of the log OR (from Table 2) for each allele of that individual.

The 247 cases of histologically confirmed NPC cases and 274 controls without NPC from the Chao Shan region of Guangdong Province were typed for HLA A and B. The results are presented in Table 1. The ORs tabulated compare individuals having at least one copy of the allele with individuals who do not have the allele. The comparison baseline is therefore slightly different for each OR. A2 and B46 significantly increase the risk, and A11 significantly reduces the risk. A33 and B58 both increase the risk, although neither increase reaches statistical significance. In addition, there are a number of other alleles which achieve nominal statistical significance: A31, A32, B13, B27, B39, B55, and B61. Of these, B13 and A31 are significant even after correcting for the number of alleles tested. In order to evaluate further the results for these additional alleles, these results were combined with the results from other studies in southern Chinese populations. A number of studies (Cui and Lin, 1982; Ou *et al*, 1985; Wu *et al*, 1989; Ooi *et al*, 1997; Hildesheim *et al*, 2002; Lu *et al*, 2003) have reported on the association of HLA alleles with risk for NPC among southern Chinese, but only the two most recent (Hildesheim *et al*, 2002; Lu *et al*, 2003) reported on the range of alleles given in Table 1. We therefore combined the results from these two studies with our results in Table 2 (as these effectively amount to a meta-analysis of such studies in the Southern Chinese). A31, B13, B27, B39, and B55 are all strongly associated with NPC risk, and it is noteworthy that all the associations are negative. The negative associations for B13 and B27 are supported by earlier results among the Chinese (Chan *et al*, 1983), which have not been included in Table 2 since they did not report on A31, B39, B55, and B61. The wide range of HLA-associated risk for NPC among the southern Chinese population can be shown by creating a risk score based on the sum of the log OR of each allele carried by each individual (see the legend to Figure 1). Figure 1 gives the distribution among cases and controls separately for this risk score. Between individuals in the lowest tertile of risk score and those in the highest, there is almost a six-fold difference in risk. This variation in risk is particularly marked for A31, with 34 out of 778 controls compared to 0 out of 734 cases carrying the allele. Even the upper limit of the 95% CI (0.11) for the OR implies a nine-fold difference in risk between those with and those without the allele. This result is seen in three different studies and so is unlikely to be an artefact. In non-Chinese-related populations, no similar HLA–NPC associations have been reported, although studies are small, due to the rarity of the disease. Among Caucasians, A31, B13, and B27 were more common in cases than in controls (Moore *et al*, 1983). In north Africans from Algeria (Herait *et al*, 1983) and Morocco (Dardari *et al*, 2001), neither B13 nor B27 conferred protection (no results were given for A31, B39, or B55).

## DISCUSSION

In summary, the results demonstrate, more clearly than previously, the following. (1) There are a number of HLA A and B alleles that are associated with low, or very low, risk, relative to the rest of the Southern Chinese population. (2) There is a wide range of HLA-associated risk dispersed across the entire population, mediated by many of the HLA A and B alleles that occur with appreciable frequency in the population. (3) No similar range and diversity of HLA-associated risk is seen in non-Chinese populations. (4) The risk associated with these HLA alleles can explain only a small part of the elevated risk in Chinese.

It would therefore seem implausible that HLA molecules themselves are responsible either for the large HLA-related variation in risk (Lu *et al*, 2003) seen only in Chinese populations, or for the high risk among the Chinese.

However, an NPC disease susceptibility (DS) mutation, which occurred after the separation of the southern Chinese and related groups from other ethnic groups (Cavalli-Sforza *et al*, 1994), in a gene closely linked to but separate from HLA A and B, could generate this pattern of risk. The ORs for each of the HLA A and B allele seen in Tables 1 and 2 would reflect the frequencies with which this DS mutation occurred in the presence of or in the absence of that allele on the same chromosome. Thus, the DS mutation would occur rarely on the same chromosome as A31, whereas it would occur more frequently on the same chromosome with B46 rather than without B46.

The fact that a single copy of A31 is associated with very low risk suggests that high risk for NPC requires an individual to be homozygous for the NPC DS allele, that is, that the DS allele has to be recessive, and only the DS homozygote confers high risk. If the NPC DS allele were dominant, then the minimum relative risk it could induce for an allele in a closely linked gene is 0.5, considerably higher than some of the risks seen in Table 2. This is clear from consideration of the extreme situation when the NPC DS gene does not occur on the same chromosome as A31. If the DS gene were dominant, then it could occur only on the trans chromosome in the presence of A31, whereas it could occur on either chromosome in the absence of A31. Furthermore, a recessive DS gene does not imply that risk for HLA alleles has to be confined to the HLA homozygotes. With a recessive DS gene, the relative risk in the HLA homozygote would be the square of the relative risk in the HLA heterozygote, in both cases relative to the risk in the absence of the HLA allele. The relative risks for heterozygotes and homozygotes are, for A2 (1.60, 2.15), A11 (0.46, 0.36), B46 (1.79, 2.48), and B58 (1.15, 3.90), in reasonable agreement with the prediction, given the small numbers of homozygotes. No cases are homozygote for any of A31, B13, B27, B39, and B55.

Support for the hypothesis of a recessive DS allele comes from the earlier sib-pair study (Lu *et al*, 1990), the results of which gave substantially greater support for a recessive rather than a dominant mode of action. The maximum likelihood estimate of the gene frequency was 0.29, and 20.9 for the relative risk of the homozygote, the 80% CI including indefinitely large values for the relative risk. These estimates, which are clearly compatible with the relative risks given in Tables 1 and 2, would suggest that approximately 10% or more of the Chinese population carries the high-risk genotype. Given the cumulative rate to age 75 of 2.2% among males in Hong Kong, the highest recorded rate in southern Chinese, it is clear that the penetrance of the homozygote genotype is unlikely to be more than 20%. The main evidence against the hypothesis of a NPC DS gene closely linked to the HLA region is the negative linkage study published earlier (Feng *et al*, 2002), based on high-risk extended families. However, with a recessive mode of inheritance, penetrance of only 20%, and a gene frequency probably in the range of 30–50%, it is unlikely that the study had sufficient power to detect such a gene. In contrast, the substantial body of data from the association studies together with the sib-pair study provide strong support to the hypothesised existence of a recessive NPC DS allele of a gene, in linkage disequilibrium with HLA A and B, and largely responsible for the excess risk of NPC among southern Chinese. This is a hypothesis that can now be tested with the availability of a dense set of SNP covering a chromosomal region of several megabases on each side of the HLA A and B genes.

## Figures and Tables

**Table 1:** Frequency distribution of HLA-A and HLA-B genes in NPC patients and controls

	**NPC cases**	**Control subjects**	**OR**	***χ* ^2^**
*HLA A allele*				
A1	0.008	0.007	1.11	0.01
A2	0.643	0.529	1.61	7.00*
A3	0.004	0.010	0.37	0.81
A11	0.380	0.572	0.46	19.23*
A24	0.291	0.321	0.87	0.54
A26	0.068	0.054	1.28	0.45
A29	0.004	0.003	1.11	0.01
A30	0.036	0.032	1.11	0.05
A31	0.000	0.040	0.00	10.11*
A32	0.024	0.003	6.80	4.17*
A33	0.246	0.200	1.31	1.60
A68	0.008	0.003	2.23	0.45
A80	0.004	0.000	0.00	1.11
				
*HLA B allele*				
B7	0.000	0.003	0.00	0.90
B8	0.000	0.003	0.00	0.90
B13	0.076	0.178	0.38	11.87*
B15	0.024	0.018	1.34	0.23
B18	0.000	0.007	0.00	1.81
B27	0.024	0.069	0.33	5.76*
B35	0.052	0.065	0.79	0.40
B37	0.008	0.007	1.11	0.01
B38	0.153	0.109	1.48	2.25
B39	0.004	0.043	0.09	8.42*
B44	0.012	0.003	3.36	1.23
B46	0.344	0.226	1.79	8.89*
B48	0.024	0.018	1.34	0.23
B51	0.113	0.105	1.08	0.08
B52	0.016	0.025	0.63	0.55
B53	0.004	0.000	0.00	1.11
B54	0.060	0.051	1.20	0.23
B55	0.016	0.058	0.27	6.25*
B56	0.024	0.018	1.34	0.23
B57	0.004	0.003	1.11	0.01
B58	0.246	0.222	1.15	0.43
B60	0.336	0.368	0.87	0.60
B61	0.080	0.032	2.59	5.71*
B62	0.105	0.098	1.08	0.06
B67	0.008	0.003	2.23	0.45
B71	0.000	0.007	0.00	1.81
B72	0.012	0.018	0.66	0.32
B75	0.068	0.087	0.77	0.63
B76	0.008	0.014	0.55	0.48
B81	0.000	0.003	0.00	0.90

* *P*<0.001,

* *P*<0.005,

* *P*<0.01,

* *P*<0.05.

**Table 2:** Odds ratios for selected HLA A and B alleles, combining data from this study and two previous studies

**Allele**	**OR**	***χ* ^2^**	***P***	**95% CI**
A2	1.81	15.31	0.0001	1.33–2.48^a^
A11	0.55	32.81	0.0000	0.45–0.68^b^
A31	0.00	33.76	0.0000	0.00–0.11^b^
A33	1.37	4.58	0.0323	1.02–1.85^c^
B13	0.50	18.24	0.0000	0.35–0.69^b^
B27	0.49	5.51	0.0189	0.25–0.92^c^
B38	1.73	6.04	0.0140	1.09–2.76^c^
B39	0.18	15.31	0.0001	0.06–0.48^b^
B46	1.69	21.99	0.0000	1.35–2.13^b^
B55	0.32	10.55	0.0012	0.14–0.68^c^
B58	1.30	4.57	0.0324	1.01–1.68^b^

a Based on this study and Lu *et al* (2003).

b Based on this study, Lu *et al* (2003) and both phases of Hildesheim *et al* (2002).

c Based on this study, Lu *et al* (2003) and Phase 1 of Hildesheim *et al* (2002).
